# STING Signaling and Skin Cancers

**DOI:** 10.3390/cancers13225603

**Published:** 2021-11-09

**Authors:** Sayaka Sato, Yu Sawada, Motonobu Nakamura

**Affiliations:** Department of Dermatology, University of Occupational and Environmental Health, 1-1 Iseigaoka, Yahatanishi-Ku, Kitakyushu 807-8555, Japan; sato-sayaka@med.uoeh-u.ac.jp (S.S.); motonaka@med.uoeh-u.ac.jp (M.N.)

**Keywords:** STING, skin cancers, IFN, epigenetics

## Abstract

**Simple Summary:**

Stimulator of interferon genes (STING) is currently recognized as a driver for anti-tumor immunity against various malignancies and is expected to enhance the anti-tumor effects. In this review, we summarized recent knowledges gained from epigenetics-mediated skin cancer development and discussed the clinical application of STING agonists in the treatment of skin cancer.

**Abstract:**

Recent developments in immunotherapy against malignancies overcome the disadvantages of traditional systemic treatments; however, this immune checkpoint treatment is not perfect and cannot obtain a satisfactory clinical outcome in all cases. Therefore, an additional therapeutic option for malignancy is needed in oncology. Stimulator of interferon genes (STING) has recently been highlighted as a strong type I interferon driver and shows anti-tumor immunity against various malignancies. STING-targeted anti-tumor immunotherapy is expected to enhance the anti-tumor effects and clinical outcomes of immunotherapy against malignancies. In this review, we focus on recent advancements in the knowledge gained from research on STING signaling in skin cancers. In addition to the limitations of STING-targeted immunotherapy, we also discuss the clinical application of STING agonists in the treatment of skin cancer.

## 1. Introduction

Immune cells circulate in the human body for protection against external stimuli, such as antigens and microorganisms, by innate immunity and antigen-specific acquired immune responses [1,2]. Tumor cells are also recognized by host immune cells, and the importance of anti-tumor immune reactions to eradicate malignancies has been identified for a long time in the clinical scenario [3]. In particular, acquired immune responses contribute to anti-tumor immunity against malignancies mediated by cytotoxic reactions to tumor cells [4,5]. In contrast, immune cells face some difficulty in exerting anti-tumor immune responses to malignant tumor cells. One of the representative reasons is that tumor cells establish a clever escape strategy from anti-tumor immunity mediated by the signaling of PD-1/PD-L1 or CTLA-4 [6,7]. Theoretically, we expect to observe a sufficient anti-tumor immune response to malignancies by immune checkpoint inhibitor treatment; however, this treatment is not perfect, and it has been unable to obtain a satisfactory clinical outcome in all cases [6,7]. Therefore, an additional therapeutic option for malignancy is currently needed in oncology.

Type I interferon (IFN) is a representative inflammatory cytokine that activates the acquired immune response mediated by antigen-presenting cells and subsequently drives cytotoxic cell expansion and activation [8,9]. Type I IFN is currently used to treat malignancies [10,11]. However, the currently used type I IFN treatment does not yield sufficient therapeutic outcomes. Therefore, a more potent type I IFN driver might have a beneficial impact on the treatment of malignancies.

Among various type I IFN-inducible candidates, stimulator of IFN genes (STING) has been recently recognized as a strong type I IFN driver and it has shown anti-tumor immunity against various malignancies [12,13]. STING is activated by external stimuli, such as viruses, and leads to the enhancement of IFN-β-mediated augmentation of immune responses. Therefore, STING-targeted anti-tumor immunotherapy is highlighted by clinicians as an enhancer of the anti-tumor effects and clinical outcomes of immunotherapy against malignancies. However, there are only a limited number of review papers in dermatology.

In the present review, we focus on recent advancements in the knowledge gained from research regarding STING signaling in skin cancers. In addition to the limitations of STING-targeted immunotherapy, we also discuss the clinical application of STING agonists in skin cancer immunotherapy in the future.

## 2. The Mechanism of STING Signaling

STING is a crucial positive immune driver that induces the production of type I IFN by triggering intracellular pathogens, such as viruses [14]. STING-mediated type I IFN promotes the activation of acquired immune responses mediated by antigen-presenting cells and enhances downstream cytotoxic immune reactions [9].

**Figure 1:**
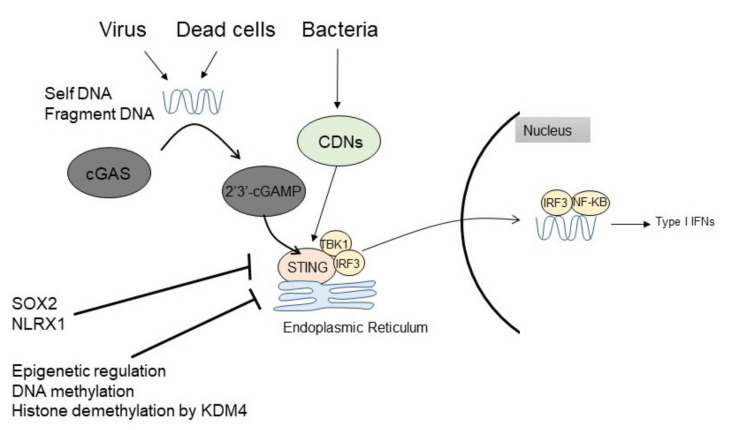
Pathway of STING signaling and anti-tumor immunity. Cytosolic DNA triggers the activation of cGAS-cGAMP-STING signaling. Cytosolic DNA is detected by a DNA sensor protein, cGAS, which enhances the synthase of 2′3′-cyclic GMP-AMP (2′3′-cGAMP). cGAMP plays as a second messenger for the activation of STING. Bacteria can also generate CDNs and activate STING independently of cGAS. STING activates transcription factors IRF3 and STAT6 through TBK1 and promotes the gene transcription of type I IFN. DNA: deoxyribonucleic acid, cGAS: cyclic GMP-AMP synthase, CDNs: cyclic dinucleotides, STING: Stimulator of IFN genes; TBK1: tank binding kinase 1; IRF3: interferon regulatory factor 3, NF-kB: nuclear factor-kappa B; IFN: interferon; SOX: SRY-box transcription factors; NLRX1: NLR Family Member X1.

**Figure 2:**
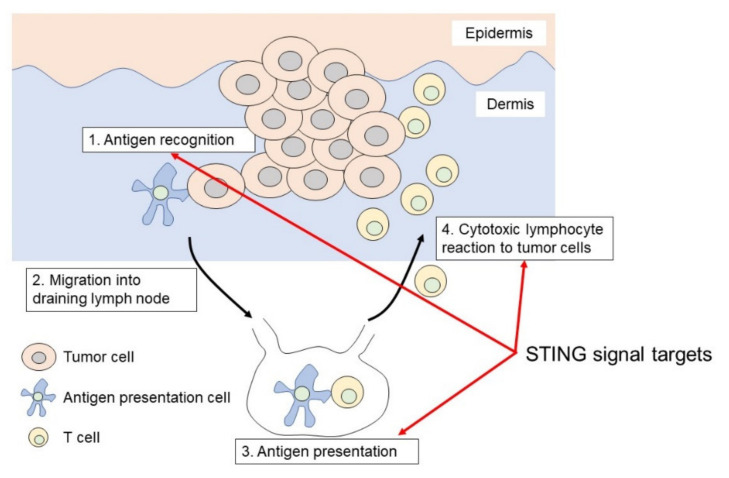
Cutaneous immune responses against tumor cells. Antigen-presenting cells, especially dendritic cells, recognize antigens on the surface of skin cancers, and migrate to draining lymph nodes to present tumor antigens to naïve T-cells for the induction of antigen-specific reactive T-cells. These tumor antigen-specific T-cells react to tumors to drive an anti-tumor immune response.

As an activation mechanism, cytosolic DNA becomes a trigger for the activation of cGAS-cGAMP-STING signaling [15,16,17] (Figure 1). Cytoplasmic DNA is a danger signal that is released from the nucleus and mitochondria, or by the induction of viruses or bacteria [14]. These released cytosolic DNAs, such as double-strand DNA (dsDNA) or single-strand DNA (ssDNA), are detected by a DNA sensor protein, cGAS, which enhances the synthase of 2′3′-cyclic GMP-AMP (2′3′-cGAMP). cGAMP acts as a second messenger for STING activation [15,16,17,18,19]. dsDNA acts upstream of STING on cGAS, while bacteria can also generate CDNs and activate STING independently of cGAS [20,21,22]. STING consists of four transmembrane domains located in the endoplasmic reticulum (ER). After cGAMP binds to STING, STING translocates to the perinuclear site and drives the downstream cascade. STING positively regulates the downstream pathway and activates the transcription factor interferon regulatory factor 3 (IRF3) and signal transducer and activator of transcription 6 (STAT6) through TANK-binding kinase 1 (TBK1). STING activates TBK1 to enhance the phosphorylation of IRF3 or STAT6, which enters the nucleus to promote the transcription of type I IFN. Type I IFN enhances antigen presentation ability and T-cell proliferation in the skin [8,9] (Figure 2). To drive anti-tumor acquired immune responses, antigen-presenting cells, especially dendritic cells, recognize antigens on the surface of skin cancers, and migrate to draining lymph nodes to present antigens to naïve T-cells for the induction of antigen-specific reactive T-cells [23,24]. Tumor antigen-specific T-cells drive anti-tumor immune responses mediated mainly by cytotoxic reactions. Therefore, these signaling effects are expected to elicit an anti-tumor immune response against skin cancers.

In addition, the STING signaling pathway plays an important role in various skin diseases. Keratinocytes are major constitutive cells in the epidermis and regulate inflammatory responses against external environmental stimuli involved in various inflammatory skin diseases [25,26,27,28]. The characteristics of the keratinocytes located in the outermost layer of the skin suggest they can be influenced by external organisms to induce inflammatory cytokine production. Cytosolic DNA induces weak inflammatory responses in keratinocytes; however, these effects are synergized with TNF-α and IL-1β [29]. Therefore, STING signaling may be involved in various inflammatory processes under certain inflammatory skin conditions. Indeed, STING signals enhance the degree of inflammation in skin diseases, such as psoriasis [30], acne [31], and hidradenitis suppurativa [32]. Therefore, the skin is expected to be influenced by STING signaling.

## 3. STING-Involved Anti-Tumor Immunity

There are recent updates in the research on STING in skin cancers. In this section, we refer to skin malignancies in the detailed molecular mechanism of STING-mediated anti-tumor immunity, such as melanoma, squamous cell carcinoma, Merkel cell carcinoma, and adult T-cell leukemia/lymphoma.

### 3.1. Melanoma

Melanoma is a malignancy derived from melanocytes that shows unfavorable clinical outcomes following current treatment [33]. Recent advancements in immune checkpoint inhibitor treatment or BRAF-mutation targeted inhibitors dramatically overcome the limitations of current treatments and yield favorable clinical outcomes [34,35,36]. However, intractable cases of these novel treatments do exist in patients with melanoma. Therefore, STING-targeted treatment is currently seen as a possible alternative or additive treatment for melanoma.

STING enhances anti-tumor immunity against melanoma mediated by IFN [37,38,39,40,41], which activates immune cells such as CD11c+ DC, CD8+ cells and NK cells in response to melanoma cells [42,43,44]. STING also upregulates MHC class I molecules, thereby enhancing the recognition or killing effect of cytotoxic T-cells [45]. A STING stimulator, cGAMP, injection into the tumor leads to the activation of STING, subsequently enhancing CD8+ cell infiltration into the tumor and suppressing tumor growth [46]. STING also enhances chemokines, such as CXCL10, CCL5, and IL-33, which also contribute to the infiltration of NK cells [47]. STING induces the production of TNF-α, which is essential for anti-tumor immunity [48].

Several studies suggest a potent additional therapeutic effect of STING signaling in combination with immunotherapy. cGAS-deficient mice showed impaired growth of B16 melanomas in response to PD-L1 antibody treatment [49]. Depletion of CD8+ T-cells or Mφ impaired the anti-tumor effects of cGAMP treatment [50]. In addition, CAR-T cell treatment eradicates tumors more effectively with STING agonists that stimulate immune responses to eliminate tumor cells [51].

STING is also responsible for the efficacy of chemotherapy in melanoma. 5-fluorouracil (5-FU) is a representative chemotherapeutic agent, and its responsiveness depends on intrinsic STING signaling in tumor cells and subsequent type I IFN production. Consistently, the deficiency of STING in tumor cells is related to the requirement of a higher dose of 5-FU to exert anti-tumor effects [52].

Radiation is one of the therapeutic options for melanoma treatment, and combination therapy with an immune checkpoint inhibitor is expected to show abscopal effects against melanoma [53]. Consistently, inhibition of pattern recognition by STING signaling negatively regulates type 1 IFN production and prevents the regression of abscopal tumors by treatment with radiation and an immune checkpoint blockade [54]. Since the NF-κB pathway supports the initiation and progression of tumors, this pathway involves the mechanisms of radiotherapy resistance of tumor cells. The deficiency of non-canonical NF-κB activates radiation-induced anti-tumor immunity mediated by the STING sensor-dependent DNA-sensing pathway, triggering DC activation [55]. Therefore, STING is also involved in resistance to radiotherapy.

### 3.2. Cutaneous Squamous Cell Carcinoma

Cutaneous squamous cell carcinoma is a skin cancer derived from epithelial keratinocytes, and its advanced form has an unfavorable clinical course because of the limited number of therapeutic options in the current treatment [56,57,58]. In addition, immune checkpoint inhibitor treatment is not widely used for treating metastatic cutaneous squamous cell carcinoma. Therefore, clinicians need novel treatment options for cutaneous squamous cell carcinoma.

STING has been identified as an intrinsic regulator of squamous cell carcinoma survival [59,60]. STING positively regulates the generation of reactive oxygen species (ROS), and STING reduction impairs DNA damage, leading to therapeutic resistance. Consistently, low STING expression in squamous cell carcinoma is associated with unfavorable clinical behavior. The pharmacological activation of STING enhances anti-tumor effects by combining DNA-damaging agents [59]. STING induces type 1 IFN and CD8+ T-cell-mediated anti-tumor immunity [61], and enhances the production of immunosuppressive cytokines and impairs the infiltration of regulatory T-cells [62].

In contrast, cytosolic DNA triggers chromosomal instability in tumor cells and enhances tumor metastasis. This depends on the activation of cGAS-STING signaling by cytosolic DNA-sensing stimulation [63].

Activated-STING increases IFN production and enhances the expression of PD-1 pathway in vivo [59]. STING is also a positive driver of chemotherapy-induced anti-tumor immunity. Combination therapy using cisplatin and cGAMP enhances the gene expression of CXCL9 and CXCL10 in tumor tissues and inhibits tumor growth [64]. STING promotes the cetuximab-induced activation of NK cells and DCs [65].

### 3.3. Merkel Cell Carcinoma

Merkel cell carcinoma is a rare cutaneous malignancy derived from neuroendocrine cells, and Merkel cells act as mechanoreceptors. Merkel cell carcinoma exhibits aggressive clinical behavior with a high mortality rate. Elderly and immunocompromised host conditions contribute to the development of Merkel cell carcinoma. Although there are a limited number of therapeutic options for Merkel cell carcinoma with distant metastasis and immune checkpoint inhibitor treatment, avelumab shows favorable clinical outcomes. Therefore, anti-tumor immunity against Merkel cell carcinoma is needed as an additional therapeutic option.

The Merkel cell polyoma virus plays an important role in the oncogenesis of Merkel cell carcinoma, and its replication and/or transcription drive an innate immune response via cGAS-STING [66]. Furthermore, STING is completely silenced in Merkel cell carcinomas [67]. STING deficiency contributes to the immunosuppressive nature of Merkel cell carcinoma. STING agonists enhance cell death in Merkel cell carcinoma in addition to DNA released by the dying cancer cells, which enhances the innate immune response and activates anti-tumor adaptive responses. Therefore, STING signaling in Merkel cell carcinoma plays an important role in anti-tumor immunity.

### 3.4. Adult T-Cell Leukemia/Lymphoma

Adult T-cell leukemia/lymphoma is a malignancy associated with human T-cell lymphotropic virus type I (HTLV-1)-infected mature CD4+ T-cells [68,69]. Adult T-cell leukemia/lymphoma is divided into four clinical groups according to Shimoyama’s classification based on the severity, number of abnormal lymphocytes, and organ involvement [69]. Skin lesions are observed in approximately 50% of adult T-cell leukemia/lymphoma patients, and the assessment of skin lesions is helpful in prognosis [70,71,72]. Although aggressive types, namely the acute and lymphoma types of adult T-cell leukemia/lymphoma, show an unfavorable clinical course [73,74,75], the chronic and smoldering types are indolent and can usually be managed by “watchful waiting” [76].

IFN is a key therapeutic target for the innate immune response to viruses. IFN-α is a standard therapeutic option for adult T-cell leukemia/lymphoma with a combination of the nucleoside reverse transcriptase inhibitor zidovudine. Tax expression, which is responsible for the development of adult T-cell leukemia/lymphoma, suppresses the induction of IFN production by cGAMP synthase plus STING stimulation [77]. STING enhances the formation of a complex of IRF3-Bax leading to adult T-cell leukemia/lymphoma apoptosis, suggesting that STING is responsible for anti-tumor activity against adult T-cell leukemia/lymphoma [78].

### 3.5. STING Anti-Tumor Effect Expected Skin Cancers

Although there have been no reports regarding the relationship between STING and its downstream cytokines, these are involved in the suppression of basal cell carcinoma [79,80,81], diffuse large B-cell lymphoma [82], mycosis fungoides [83,84,85,86,87,88,89,90], and Sézary syndrome [83,84,85,91,92,93,94,95]. Therefore, these cutaneous malignancies are expected to have a beneficial effect on STING-mediated anti-tumor effects.

#### 3.5.1. Basal Cell Carcinoma

Basal cell carcinoma is a malignancy arising from the epidermal basal cells [96]. Although basal cell carcinoma shows local invasion, metastasis is a rare event. There are some drugs, such as a hedgehog signaling pathway inhibitor, but these have not achieved successful outcomes. Due to the limited number of therapeutic options for metastatic basal cell carcinoma in such rare cases, a therapeutic candidate for anti-tumor immune responses against basal cell carcinoma has been required for a long time.

IFN-α injection into the tumor is effective in basal cell carcinoma [79]. IFN-α induces the expression of Fas in basal cell carcinoma leading to apoptosis of the tumor [80], suggesting that type I IFN plays an important role in the regulation of basal cell carcinoma. In addition, a previous study reported that IFN-γ increased in spontaneously regressing basal cell carcinoma, suggesting that cytotoxic immune reaction-mediated Th1 might play a pivotal role in the regulation of basal cell carcinoma [81]. STING drives the downstream immune reaction mediated by CD8+ T-cells, which produce an abundance of IFN-γ, and is expected to have beneficial therapeutic effects.

#### 3.5.2. Cutaneous Lymphomas

Diffuse large B-cell lymphoma is a malignancy of B-cells and it is a common type of non-Hodgkin cutaneous lymphoma [97]. Cutaneous diffuse large B-cell lymphoma is commonly observed in the extremities and clinically appears as solid nodules or tumors in the skin. In addition to the specific therapeutic approach for diffuse large B-cell lymphoma targeting CD20 cell surface markers, immunotherapy is currently highlighted as an alternative treatment option for clinicians.

Although there have been no reports regarding STING and diffuse large B-cell lymphoma, IFNs have been used for treatment. Among IFNs, IFN-β shows more potent direct suppressive effects on tumor cell growth by inducing apoptotic cell death following DNA damage, caspase-3 activation, and the annexin V binding effect [82]. Therefore, STING-mediated IFN is expected to show anti-tumor effects on diffuse large B-cell lymphoma.

Mycosis fungoides are malignant tumors of T lymphocytes with epidermotropism in the skin [68,98,99,100,101]. Mycosis fungoides exhibits an indolent clinical course with a slow advancement starting from patches to plaques and eventually developing into nodules/tumors as a more infiltrated form [68]. The prognosis of mycosis fungoides is closely related to the clinical stage, skin involvement, and systemic organ involvement [68].

Several studies have shown the therapeutic effects of IFN-α on mycosis fungoides [83]. IFN-α enhances anti-tumor effects in combination with chemotherapy, phototherapy, and anti-CCR4 antibody treatment [84,85,86]. Topical IFN-β showed therapeutic potential for mycosis fungoides and showed rapid tumor resolution [87]. Furthermore, intratumoral IFN-γ administration has a therapeutic effect on mycosis fungoides [88,89,90].

Sézary syndrome is a T lymphocyte-derived malignancy characterized by erythroderma as a clinical manifestation [98,99,102]. Although STING-mediated anti-tumor effects have not been reported in patients with Sézary syndrome, the downstream of STING signaling IFNs have also been investigated as a therapeutic option for Sézary syndrome. IFN-α is effective for Sézary syndrome [91] and the combination of IFN-α and chemotherapy or phototherapy shows more potent therapeutic effects [83,84,85,92,93,94]. IFN-γ also enhances cytotoxic activity against tumor cells in Sézary syndrome [95]. Therefore, these findings suggest that STING-mediated anti-tumor immune reactions might be beneficial for the treatment of Sézary syndrome.

Other effects of IFNs are those against tumor and anti-tumor immune responses. IFN-α/β enhances immunoglobulin production by B-cells. IFN-γ enhances NK cell activity, suppresses tumor growth, and reduces IL-4 production in patients with mycosis fungoides. Therefore, STING-targeted treatment is expected to show the effect of these downstream cytokines on mycosis fungoides.

## 4. STING Strategy for Skin Cancers

To obtain a more potent anti-tumor effect mediated by the STING signal, some modifications of the STING stimulator have been investigated. Cationic liposomes with varying surface polyethylene glycol (PEG) are used to encapsulate cGAMP to facilitate its cytosolic delivery, and antigen-presenting cells improve the cellular uptake of cGAMP and pro-inflammatory gene induction [103]. Biodegradable poly(beta-amino ester) (PBAE) nanoparticles to deliver CDNs for STING agonists synergize with checkpoint inhibitors and have strong potential to enhance cancer immunotherapy [104]. cGAMP encapsulated in lipid nanoparticles conjugated with mannose (LP-cGAMP) was designed for delivery to DCs. LP-cGAMP potently drives STING-mediated inflammatory reactions and subsequently enhances CD8+ T-cell infiltration. Consistently, STING activation upregulates PD-L1 on tumor cells and has a beneficial impact on the anti-tumor immune response in B16F10 and BRAF-mutated murine melanoma animal models [105]. Microfabricated polylactic-co-glycolic acid (PLGA) particles encapsulated a STING agonist that triggers anti-tumor immune reactions to suppress tumor growth and have a beneficial impact on their survival. STING agonist-loaded microparticles improve the response to immune checkpoint blockade therapy [106].

In addition, several novel STING agonists have been developed for melanoma treatment. Manganese potentiates the anti-tumor immune response as a STING agonist [107]. Dimeric aminobenzimidazole (diABZI) is another STING agonist that promotes tumor cell death in melanoma in combination with BRAF inhibitors [108]. Cytomegalovirus (CMV) acts as a STING agonist in tumors. CMV-infected tumors in STING-deficient mice show no additional immune reactions, such as macrophage and CD8+ T-cell infiltration and decreased inflammatory cytokine and chemokine production [109]. Two highly potent cyclic dinucleotide STING agonists, IACS-8803 and IACS-8779, were developed as STING agonists and enhanced systemic anti-tumor responses in a B16 murine model of melanoma [110]. Haspin kinase (HASPIN) is related to the regulation of STING signaling; therefore, HASPIN inhibition reduces proliferation of the tumor cells by the activation of the STING pathway and STING-dependent type I IFN and decreased Treg [111]. ADU-S100 is a STING agonist [112] and it has anti-tumor effects in cervical cancer [113], pancreatic cancer [114], esophageal adenocarcinoma [115], and prostate cancer. Therefore, this analog is expected to have anti-tumor effects against cutaneous malignancies.

Combination therapy with STING has been proposed for the treatment of melanoma. The phagocytosis checkpoint of signal regulatory protein α (SIRPα) and STING in antigen presentation cells enhance the anti-tumor immune response [116]. STING signaling activates antigen-presenting cells to induce the activation of CD8+ cells. In addition, a nanoparticle combined with TLR9, STING, and RIG-I with a melanoma-specific peptide enhances the anti-tumor immune response [117].

There are several reports on STING-targeted therapeutic options. SOX2 acts as a STING inhibitor. SOX2 enhances STING degradation in an autophagy-dependent manner and subsequently suppresses the production of type I IFN. Consistently, SOX2 supports squamous cell carcinoma cell growth by suppressing anti-tumor immunity. A combination of a STING agonist with anti-PD-L1 antibody treatment enhances the tumor-specific cytotoxic lymphocyte reaction, thereby improving survival [118]. NLRX1 is a mitochondrial NOD-like receptor that enhances the NF-κB and JNK pathways through the production of ROS. NLRX1 promotes HPV16 E7-potentiated STING turnover. Consistently, NLRX1 depletion activates type I IFN-dependent T-cell infiltration profiles and tumor control [119].

## 5. Epigenetic Modification

Epigenetic changes are chemical modifications of DNA and DNA-binding histones, which can modulate chromatin structure and gene transcription by exposure to environmental stimuli without changing DNA sequence information [120].

STING signaling is not suitable for melanoma cells in terms of long-term tumor development because it triggers anti-tumor immunity. As one of the strategies for melanoma development, STING is suppressed in melanoma cells by epigenetic silencing of STING and cyclic GMP-AMP synthase [121], possibly due to suppressed DNA hypermethylation in melanoma cells. The presence of considerable CpG islands within the STING and cGAS promoter regions contributes to the epigenetic regulation of STING in melanoma cells. Indeed, a demethylase, 5AZADC, treated melanoma cells following dsDNA stimulation and showed recovered IFN-β and CXCL10 production in melanoma cells suggesting that DNA methylation might play a role in the regulation of STING in melanoma. In addition, as another mechanism to suppress STING signaling, STING signaling may be commonly suppressed in a greater variety of tumors due to loss-of-function mutations or epigenetic silencing of the STING/cGAS promoter regions [122].

In the STING modification of epigenetic changes in squamous cell carcinoma, KDM4A inhibition activates immune responses to squamous cell carcinoma and enhances the therapeutic potential of anti-PD-1 antibody treatment for squamous cell carcinoma. KDM4A is a histone demethylase that targets histone H3 lysine 9 trimethylation (H3K9me3) and is associated with the development of squamous cell carcinoma growth and metastasis. KDM4A inhibition activates cGAS-STING signaling in tumor cells. Consistently, KDM4A inhibition with the combination of anti-PD-1 antibody treatment suppresses tumor growth and metastasis [123], suggesting that epigenetic modification of STING signaling mediated by KDM4A histone demethylation might positively drive anti-tumor immunity.

## 6. Limitations and Disadvantages of STING-Mediated Anti-Tumor Immunity

Although STING is anticipated as an additional immunotherapy treatment against skin cancers, downstream cytokines of IFNs are needed to boost immunotherapy against cancers. However, IFN-treated patients did not exhibit the dramatic therapeutic potential of IFN-β during the treatment of skin cancers. Therefore, it seems that STING might not show satisfactory clinical outcomes in all cases as a driver of the anti-tumor immune reaction to skin cancers.

The current administration of IFN-β transiently increases its concentration in the human body. Therefore, sustainable IFN-β production from the host might have more potent anti-tumor effects against skin cancers. As so, STING agonist administration can drive IFNs; however, the treatment itself might limit the transient production of IFNs.

Because various cells can react with IFNs, it is important for IFNs to react to anti-tumor immune cell-specific or tumor sites to obtain the maximum effects of STING. Therefore, systemic STING agonists might result in the same pitfall as systemic IFN treatment or subcutaneous injection of IFNs. A recent study identified that IFN-independent STING signaling enhances autophagy [124], which can enhance anti-tumor immune responses. In addition, STING signaling triggers DNA damage response to tumor cells [125,126] and MHC class I expression in tumor and immune cells [127]. These additive non-IFN-independent effects of STING might also contribute to the development of anti-tumor responses.

In addition, STING signaling activation might result in the enhancement of autoimmune reactions, as seen in immune checkpoint inhibitor treatment. STING activation has been observed in various autoimmune diseases, such as rheumatoid arthritis [128] and dermatomyositis [129]. Therefore, clinicians should keep in mind that STING-mediated anti-tumor therapy might show immune cell-mediated adverse event (irAE) reactions during treatment [130]. Furthermore, combination therapy with immune checkpoint inhibitors will be a therapeutic option for malignancies; however, a more potent risk of severe irAEs will need to be discussed in the future. As a strategy against STING-triggered irAEs, corticosteroids might be the first approach to suppress the symptoms of adverse reactions of STING-mediators. In addition, downstream cytokine suppression might also be a candidate for the treatment of STING-mediated irAEs. Anti-IFN antibodies are currently used for various treatments [131]; therefore, these agents may also be used for STING-mediated irAE treatment.

## 7. Conclusions

STING is anticipated as a therapeutic candidate for the treatment of an advanced form of skin cancer; however, it remains unclear whether STING signaling is involved in the regulation of tumor development. On the other hand, STING-targeted treatment might have disadvantages, possibly autoimmune-mediated systemic reactions, indicating that clinicians might keep in mind this advantage of STING-targeted treatment for skin cancers. Furthermore, STING-mediated inflammation may also contribute to the development of another malignancy [132]. Given that there is a large frontier field regarding STING-mediated anti-tumor function in skin cancers, further investigation is required to clarify the detailed roles of STING in skin cancer.
